# Scientific Value of the Sub-Cohort of Children in the World Trade Center Health Registry

**DOI:** 10.3390/ijerph191912461

**Published:** 2022-09-30

**Authors:** Robert M. Brackbill, Emma Butturini, James E. Cone, Ayda Ahmadi, Robert D. Daniels, Mark R. Farfel, Travis Kubale

**Affiliations:** 1 World Trade Center Health Registry, Division of Epidemiology, New York City Department of Health and Mental Hygiene, New York, NY 11101, USA; 2 World Trade Center Health Program Division, National Institute for Occupational Safety and Health, U.S. Centers for Disease Control and Prevention, Atlanta, GA 30329, USA

**Keywords:** 9/11 disaster, children exposed to disasters, WTC cohorts

## Abstract

The World Trade Center Health Registry (WTCHR) was established in 2002 as a public health resource to monitor the health effects from the World Trade Center (WTC) disaster. We evaluated the representativeness of the WTC youth population (<18 years on 11 September 2001) by comparing the distributions of age, gender, race/ethnic groups, and income to 2000 census data for the matched geographic area, including distance from disaster. There were 2379 WTCHR enrolled children living in Lower Manhattan south of Canal Street on 11 September 2001, along with 752 enrolled students who attended school in Lower Manhattan but were not area residents. The WTCHR sub-group of children who were residents was similar to the geographically corresponding census population on age and sex. Black and Hispanic children are moderately overrepresented at 0.9% and 2.4% in the WTCHR compared to 0.8% and 1.7% in census population, respectively, while lower-income households are slightly under-represented, 28.8% in the WTCHR and 30.8% for the corresponding census information. Asian children appear underrepresented at 3.0% participation compared to 6.3% in the census. While the demographics of WTCHR youth are somewhat skewed, the gaps are within expected patterns of under-representation observed in other longitudinal cohorts and can be effectively addressed analytically or through targeted study design.

## 1. Introduction

The WTC Health Registry (WTCHR), established in 2002, was conceived and planned by the New York City (NYC) Department of Health and Mental Hygiene in collaboration with the Agency for Toxic Substances and Disease Registry (ATSDR). Housed in a dedicated research unit within the New York City Department of Health and Mental Hygiene’s Division of Epidemiology, the WTCHR is an essential public health resource for understanding of the short- and long-term (20+ years) physical and mental health conditions caused by the terrorist attacks on 11 September 2001 (9/11). Information provided by the WTCHR to WTC responders, WTC survivors, researchers, and policymakers informs 9/11 health care and helps those affected make informed decisions about their health. The WTCHR has been funded by the National Institute of Occupational Safety and Health (NIOSH) since May 2009.

The primary goals of the WTCHR include:

- Identifying individuals present during and in the aftermath of the disaster;
- Assessing the occurrence of various health effects among survivors;
- Following participants to determine if the occurrence of health effects changes over time;
- Generating hypotheses for future studies;
- Providing health education and other information related to the aftermath of the disaster to reduce future morbidity and mortality and inform public policy [1].

In summary, the WTCHR was created to gather information on the burden, frequency, diversity and course of physical and mental health conditions related to the 11 September 2001 attacks on the WTC Towers in NYC and serve as a robust source of information to support further 9/11-related research [1].

Recruitment targeted responders (e.g., firefighters, police, and rescue and recovery workers) and non-responding persons (e.g., residents, building occupants, and passers-by) who were believed to be at greatest risk of direct exposure to major hazards from the disaster (e.g., dust cloud, falling debris, indoor settled dust, and psychological trauma). Key exposure groups were identified and used to estimate the eligible population using census, employment, public and private school enrollment, and other public data sources [2]. This exposure-based approach aided efforts to examine whether hazards associated with the WTC disaster were linked to adverse health conditions in the 9/11-exposed population. Although the principal hazardous area was the immediate perimeter of the disaster site (generally defined as South of Chambers Street in Lower Manhattan), the northern geographic boundary was established at Canal Street, which had previously defined an area for assessment of outdoor and indoor exposures in lower Manhattan [3,4].

WTCHR enrollment began 12 September 2004, focusing on several highly exposed eligibility groups:

- **Workers and volunteers** involved in rescue, recovery, or clean-up at the WTC site any time from 11 September 2001, through 30 June 2002, and/or in debris handling at the Staten Island Landfill any time from 12 September 2001, through 30 June 2002.
- **Residents** as of 11 September 2001, at addresses located south of Canal Street.
- **Students and school staff** enrolled/employed in schools or daycares south of Canal Street on 11 September 2001; and
- **Building occupants, people in transit, and pedestrians, employees, visitors, and passers-by** present south of Chambers Street on 11 September 2001 [2,5,6].

Outreach for recruitment was accomplished through community engagement and media campaigns designed to inform the public and promote WTCHR participation. In addition, lists of potentially eligible Lower Manhattan residents were obtained via purchase from Genesys Sampling Systems, and three additional lists were received from tenant organizations [2]. According to the 2000 census, over 30% of adult residents were parents, and thus outreach to residents also involved encouraging them to enroll their children by proxy. Eligible children younger than 18 years at the time of the enrollment interview were enrolled by their parents or guardians. Persons aged 18 years and older at the time of enrollment were enrolled via direct contact from a list or by contacting the WTCHR themselves for self-enrollment [1,2].

Youth outreach efforts involved contacting school officials and parent-teacher organizations located south of Canal Street to deliver information promoting participation in the WTCHR. The NYC Department of Education (DOE) also sent letters to parents of 12,000 students enrolled in public schools in lower Manhattan. Individual principals also agreed to provide recruitment letters that students took home. Nine private schools provided lists of students which were used for recruitment. Midway through enrollment, an expert panel on registries was convened to validate and advise on ongoing recruitment efforts [7].

The enrollment strategy was designed to maximize the number of participants from each exposure group, while ensuring equal representation within exposure groups (i.e., comparable proportions of registrants per group by demographic categories). The affected population was estimated to be 410,761 using the same criteria for inclusion in the population denominator as for the WTCHR [6]. In this affected population, there were approximately 14,000 children younger than 18 years on 11 September 2001, not including an unknown number of passersby on that morning. Given the lack of availability of lists for recruitment of some eligible groups (e.g., the large numbers of passers-by, people in transit, and people who occupied non-WTC buildings), enrollment heavily relied on self-identification. Potentially eligible persons identified via lists and self-identification were contacted for eligibility screening, followed by an enrollment interview for those confirmed to be eligible and willing to participate. In total, there were 115,312 persons of those potentially eligible (N = 120,272) successfully contacted (95.8%), resulting in 71,424 registrants at the close of enrollment in November 2004 [8]. Among them were 3247 persons under the age of 18 on 11 September 2001, including 2379 residents and 752 non-resident students enrolled in schools south of Canal Street. This group comprised the base for research examining health conditions among the 9/11 youth population.

Heightened concern regarding the long-term effects of youth exposure to 9/11 as this population progresses into adulthood has led to scrutiny of existing information for research. This report summarizes recruitment procedures and the demographic composition of the WTCHR youth subcohort to evaluate the representativeness, sample size and length of follow-up which are key elements of scientific value for research on adverse health outcomes among youth related to 9/11 exposure. The research aim thus was to assess the degree to which the children enrolled in the WTCHR were representative of the population at risk. Representativeness is key to ensuring a viable subcohort for future research examining health conditions in this potentially vulnerable population.

## 2. Materials and Methods

### 2.1. Data Sources

WTCHR data from the Wave 1 survey provided demographic information on age, sex, race/ethnicity, and household income on 11 September 2001. Residential and school data were linked with the main WTCHR data to indicate the census tract location during 2001 and the source of recruitment (self- vs. list-identified). Public and private school data for the 2000–2001 school year are publicly available from the National Center for Education Statistics (NCES). Public school enrollment and staff data were obtained from the NCES’s Common Core of Data (CCD), and private school data came from the Private School Survey (PSS) [9,10]. Additionally, information for preschools and daycare centers came from the NYC Bureau of Day Care. The 2000 Census, which was closest census time-wise to the recruitment of residents for the WTCHR, provided the total number of residents by age, sex, and race/ethnicity within each census tract [11,12,13]. A separate source of census data, focused on economic characteristics, was used for income in 1999 by census tract [12].

### 2.2. Analysis

**Figure 1:**
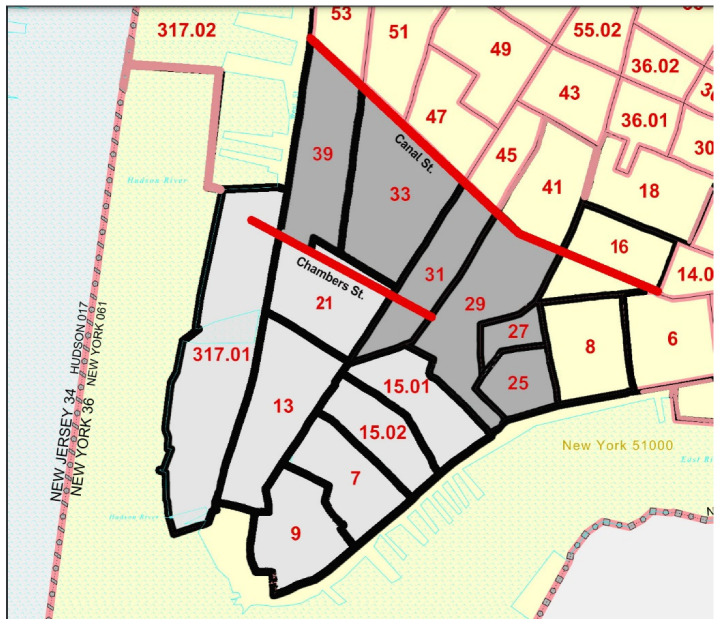
Census tract outline map for Lower Manhattan outlining census tracts for Group 1 and Group 2, New York City in 2000.

The analytical methods consisted of first defining variables to indicate whether a child aged <18 years was a resident or a student enrolled in a school on 11 September 2001 south of Canal Street in Lower Manhattan, yielding three youth groups: non-student residents, resident students, and non-resident students. Further categorization of residents resulted in two priority groups which were thought to be the most highly exposed due to proximity to the WTC disaster site. Based on this, recruitment efforts had focused first on residents who lived South of Chambers Street (Group 1), and then those who lived between Chambers Street and Canal Street (Group 2) (Figure 1). We assigned enrollees to Group 1 if they lived in census tracts 7, 9, 13, 15.01, 15.02, 21, 317.01 or Group 2 if they lived in census tracts 8, 16, 25, 27, 29, 31, 33, 39. For the analysis, all residents are restricted to the census tracts included in Group 1 and 2 (N = 12,861). The number of WTCHR residents was the sum of residents whose address could be assigned to either census tract-based group. Twelve percent (12%) of 14,656 residents lived in zip codes that included multiple census tracts and could not be assigned to a specific census tract because of insufficient address information. Because Census Tract 16 spans the border of Canal Street and only includes 4.2% of WTCHR residents, it was excluded from the census population.

Moreover, the source of enrollment was described for residents of all ages, the overall youth in the WTCHR and the three youth groups (non-student residents, resident students, and non-resident students) located south of Canal Street. Sources of enrollment include being contacted using contact information from a list (e.g., Genesys list of residents in the year 2000) or self-identifying, defined as seeking enrollment by pre-registering through a website or calling an 800 number. Children (younger than 18 years at time of enrollment interview), however, were enrolled indirectly by proxy interview by their parents or guardians, who were either contacted from a list or self-identified [1].

The additional analysis involved computing the proportional representation of age groups (0–4, 5–9, 10–14 and 15–17), race/ethnic groups (White, Black, Hispanic, and Asian), and sex using matched demographic categories of census population counts for the same specified census tracts and age as the WTCHR. The census household income (<$25,000, $25,000 to $49,999, $50,000 or more) was computed by multiplying the percent of households in each income group by the total population of Groups 1 and 2. The WTCHR household income groups were derived from the reported income in 2002. For the income analysis, only non-missing data were included for the WTCHR household income (N = 10,775).

Representativeness of the youth enrollees was assessed by comparing the distributions of age, sex, race/ethnic groups, and income to 2000 census data for the matched geographic area. Because the census did not provide household income specifically by children’s age, the income comparison was between the WTCHR resident household income for all ages and the comparable census data.

Complete representativeness would be indicated if the distributions were identical for the WTCHR and the census. We assessed whether any deviation in demographic distribution was non-representative by using the Chi square statistic with significance determined at alpha < 0.05. A non-significant Chi square indicates comparability between WTCHR and census distribution or representative. We also performed stratified comparisons by enrollment priority groups to assess whether those who lived in the more highly exposed areas were representative of the underlying census population in those locations. In addition, figures were included in the results to highlight the similarities and differences between the WTCHR proportions and the census.

## 3. Results

### 3.1. Overall Youth Enrollment

Media and advertising campaigns were successful in encouraging residents and parents to self-identify; 9306 (72% of the enrolled residents) south of Canal Street self-identified, 1759 of whom were resident children (Table 1). Additionally, there were 842 non-resident students under the age of 18 on 11 September 2001 who were self-identified (either by parent proxy or self-enrolled) for enrollment in the WTCHR, of whom 76% were public school students. Only about 6% of resident children were recruited via lists, although the cooperation rates for persons contacted from lists was 61% for those in Group 1 and lower for Group 2 at 45% [6].

The enrollment rates of youth living below Canal Street, including those unassigned to census tracts, for ages 0–4, 5–9, and 10–14 years (31.3%, 31.1%, and 29.2%, respectively) surpassed the overall WTCHR enrollment rate of 17%. Although the enrollment rate for the oldest age group, 15–17, is notably lower than the other age groups at 17.4%, it is similar to the overall rate. It can be noted that these proportions will likely remain consistent in this population over time, given that 90% of former children successfully contacted for consent to date have agreed to remain in the WTCHR as adults.

The enrollment rate was 47.2% for Hispanic youth in contrast to 13% for Asian youth. Enrollment rates for male and female youth was also similar at 28.9% and 29.3%, respectively.

### 3.2. Comparisons to Census

#### 3.2.1. Household Size

The mean number of persons in a household for WTCHR residents south of Canal St was 2.5, which is very similar to mean in the underlying population of 2.4, with 9533 households in the WTCHR versus 24,040 according to census [6]. This suggests that families included in the WTCHR are representative of family size in the underlying population.

#### 3.2.2. Age

**Figure 2:**
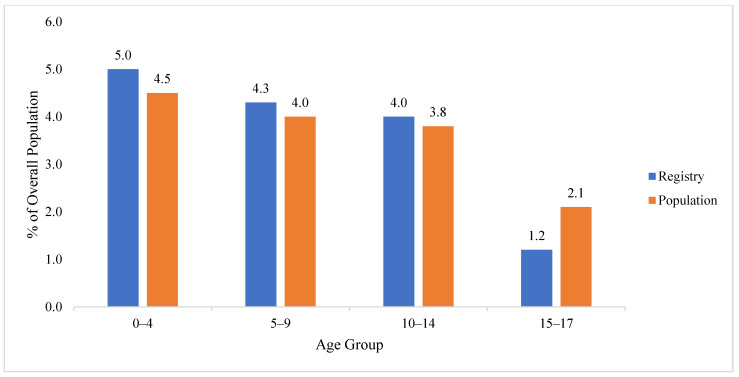
Comparison of proportions by age group of WTCHR (Registry) children to population south of Canal Street in Lower Manhattan in 2000.

Overall, the proportion of WTCHR youth aged 0–4, 5–9, and 10–14 years was similar to the age distribution in the census, but youth aged 15–17 years were somewhat underrepresented in the WTCHR (Table 2, Figure 2). Specifically, there was a significant difference (non-representative) in the proportion of youth < 15 living below Chambers Street in the WTCHR compared to census data (*X*^2^ = 14.5, *p* = 0.0007). However, there was no significant difference (representative) in the proportion of youth < 15 living between Chambers and Canal Street (*X*^2^ = 3.52, *p* = 0.17) and overall (*X*^2^ = 0.86, *p* = 0.65).

An age distribution difference between the WTCHR and census for youth 0 to 15 years of age signified an over-representation (e.g., 5.0% for WTCHR vs. 4.5% for census for 0–4; 4.3% vs. 4.0% for 5–9 and 4.0% vs. 3.8% for 10–14) of those children living South of Canal Street at the time of 11 September 2001. A greater overrepresentation of two youngest age groups occurred for enrolled children living between Canal and Chambers Street compared to those living south of Chambers Street. It is important to note that although there was a significant difference in the distribution by age of youth younger than 15 years of age south of Chambers Street, those aged 5 to 14 years appeared to be over-represented (3.6% for WTCHR vs. 3.1% for census for 5–9 and 2.7% for WTCHR vs. 2.0% for census for 10–14).

#### 3.2.3. Race/Ethnicity

**Figure 3:**
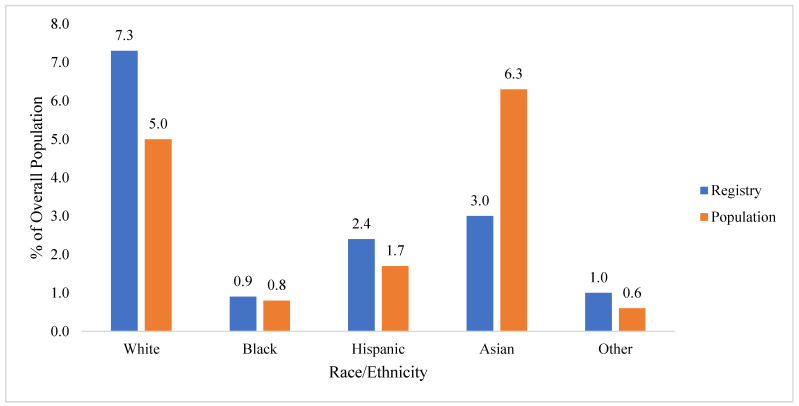
Comparison of proportions by Race/Ethnic groups of WTCHR (Registry) resident children to population south of Canal Street in Lower Manhattan in 2000.

White, Black, Hispanic, Asian, and other (e.g., multiple endorsement of race/ethnic membership) youth were unevenly distributed among residents under 18 years compared to the corresponding census population. It is notable that there were higher proportions of Black and Hispanic youth who lived south of Canal Street enrolled in the WTCHR compared to the census data (e.g., 0.9% vs. 0.8% for Blacks and 2.4% vs. 1.7% for Hispanic), while Asian youth were underrepresented overall (Table 3, Figure 3).

There was a significant difference in the distribution of race/ethnicity groups living in both areas south of Canal Street and overall (*X*^2^ = 360.25, *p* < 0.0001). The low proportion of Asian youth and relatively high proportion of youth identified as other in the WTCHR may have skewed the overall comparison, so another analysis was performed limited to White, Hispanic and Black youth. However, significant differences did persist when comparing the proportion of White, Black and Hispanic WTCHR youth living below Canal Street to the census, given the slight over-representation of each of these groups (*X*^2^ = 6.41, *p* = 0.041).

#### 3.2.4. Sex

**Figure 4:**
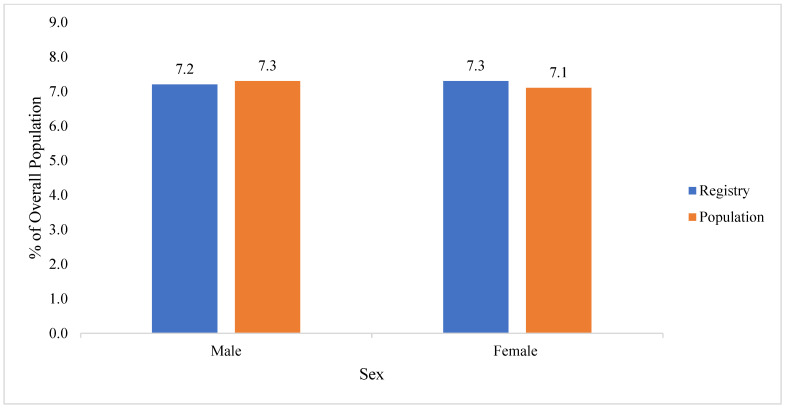
Comparison of proportions by sex of WTCHR (Registry) resident children to population south of Canal Street in Lower Manhattan in 2000.

The proportion of males and females in the WTCHR youth population living below Chambers Street (*X*^2^ = 0.11, *p* = 0.74), between Chambers and Canal Street (*X*^2^ = 2.47, *p* = 0.12), and overall (*X*^2^ = 1.29, *p* = 0.26) was not significantly different from the underlying census population (Table 4, Figure 4).

#### 3.2.5. Income

**Figure 5:**
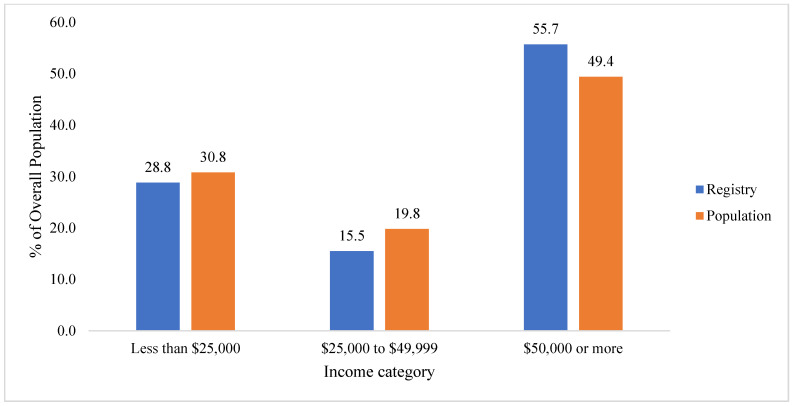
Comparison of proportions by household income of WTCHR (Registry) residents to population south of Canal Street in Lower Manhattan in 2000.

Comparing the household income distribution of enrollees with census data South of Canal Street, the Chi square indicated that the distribution by income significantly differed between the WTCHR and the census tract population (*X*^2^ = 171.92, *p* < 0.0001). The lowest income households (<$25,000 income) were similar; however, the proportion of in the WTCHR was less in the $25,000 to 49,999 household income category and more in the $50,000 or more category compared with the census tract population (Table 5, Figure 5).

## 4. Discussion

### 4.1. WTCHR Child Cohort in Context

The WTCHR child cohort is unique in several ways from other studies on children who were exposed to disasters, particularly the 9/11 disaster. First, the WTCHR used a relatively atypical census sampling frame wherein the entire defined disaster population was eligible. In a review of disaster research on children, there were no other census-based 9/11 children studies identified [14]. Second, the sample size of the WTCHR children exceeded that of most other 9/11 children disaster studies, with the exception of a series of cross-sectional studies based on self-reported information collected from a New York City wide sample of 8236 public school attendees enrolled in 4th to 12th grade [15,16,17,18]. That study data did not include information necessary for continued follow-up. Another widely cited set of children’s studies were based on 817 students from two Jewish parochial high schools in Manhattan and thus did not represent any particular geographic area or 9/11 exposure group [19].

Other notable attributes of the WTCHR children’s data are that, unlike the WTCHR, the six other longitudinal studies cited generally had small samples and relatively short follow-up [14]. For instance, samples ranged from 45 bereaved children to a US probability sample of 395 parents of children from 4 to 18 years old [20,21]. One study in this group conducted repeated interviews of 131 London school children and 20 of their parents or another of 116 New York City suburban high school students [22,23]. Most of these studies conducted second interviews less than a year after 11 September 2001. In contrast, the WTCHR’s cohort has 2379 children who lived in a highly exposed geographical area and another 753 students enrolled in schools in the highly exposed zone, who have been repeatedly surveyed over an 18-year period since their enrollment.

### 4.2. Is the WTCHR Youth Cohort Representative?

This evaluation of the WTCHR’s youth cohort has indicated that the enrolled children are representative of the population at risk on demographic attributes including age, sex, and to some extent income and race/ethnicity. A representative sample of children was obtained despite WTCHR children being nine times more likely to be self-identified than contacted from lists, whereas self-identification made up 70% of enrollments in the overall WTCHR. This indicates that parents primarily self-identified children in their families. Given the obstacles experienced in obtaining lists for the eligible youth population, the high level of parental self-identification is indicative of the success of recruitment efforts in persuading adults to enroll and have their children participate in the WTCHR. Furthermore, the relatively high cooperation rate of families contacted based on lists indicates that a high proportion of groups who did not self-identify are represented on the WTCHR.

Using census data for the matched geographic area, WTCHR enrollees younger than 15 years on 11 September 2001 are representative of the age and sex distribution in the census population. The reason for underrepresentation of youth aged 15–17 years is not clear. Among likely explanations, they were 18 years or older at the time of enrollment; therefore, requiring self-enrollment, which might not have been a priority for young healthy adults. Furthermore, this group may have relocated outside of the focused recruitment area because of college, military, or employment elsewhere.

In contrast to age and sex, all other characteristics differed among WTCHR youth compared to the youth census population. Black and Hispanic children were slightly overrepresented, while Asian children appear underrepresented. Despite extensive outreach efforts (e.g., collaborating with local community organizations, distributing pamphlets to apartment complexes, contacting local schools, and offering interviews in different languages), inaccessibility and other factors likely inhibited contacting and interviewing Asian residents at time of enrollment on the WTCHR. Still, the sample size of Asian/American youth may prove sufficient for research purposes, including building upon existing research of Asian/Americans in the 9/11-exposed population who were exposed at ages older than 18 years [24,25]. Data comparing income of households suggest that the lowest income group (<$25,000) closely matches the census proportion (28.8% for WTCHR vs. 30.8% for census), while the WTCHR had a higher proportion of incomes $50,000 or more compared to the census population (55.7% vs. 49.4%, respectively). Since health outcomes among low-income youth may be of interest in the future, the sample size of this sub-group could be used to support further research.

### 4.3. Is the WTCHR’s Youth Cohort Sufficiently Large to Detect Health Associations?

Given the variety of exposed populations and exposure types captured, and the intensive list-building and outreach efforts done in the context of significant disruption and displacement in lower Manhattan in the early post-9/11 years, obtaining a level of participation of 25% of residents south of Canal Street and 30% of all eligible persons after excluding passersby and building occupants can be viewed as a major scientific and logistical achievement [6]. Moreover, these efforts have culminated in making the WTCHR the largest post-disaster WTCHR in US history [6]. In terms of youth enrollment, the enrollment rate for youth under the age of 14 years is even higher at about 30%, which is indicative of the success of targeted efforts to recruit younger age groups. For comparison, a web-based survey of 987 adolescents randomly selected from a household panel achieved 41% completion within 2 weeks after 11 September 2001. However, 7 months after 11 September 2001 only 110 (11%) adolescents and 144 parents completed the follow-up [26].

The WTCHR provides the largest youth population available for 9/11-related research. The utility and efficacy of the youth cohort for research is supported by numerous published WTCHR studies of health conditions among 9/11-exposed children, including 12 focused solely on youth, 9 including children and other groups, and 9 external researcher publications on WTCHR youth enrollees recruited into their 9/11-related studies. WTCHR youth studies have yielded statistically significant results based on smaller samples of the children’s subcohort included in pediatric follow-up studies [27]. The WTCHR has provided data and/or facilitated recruitment for most young adult (<18 years old on 11 September 2001) projects funded by WTC Health Program [28]. In summary, the cohort of 3247 children in the WTCHR has sufficiently supported research on physical, mental, and behavioral outcomes in children and young adults to date.

The WTCHR continues to support new avenues of inquiry related to the long-term health effects of 9/11 exposure among youth, including supporting more recent research projects examining the health outcomes of older individuals who were under 18 years old on 11 September 2001. Two external researchers (Drs. Trasande and Hoven) were awarded multiple federal research grants by the WTC Health Program, housed within CDC/NIOSH, following a rigorous scientific review and scoring process to recruit subsets of WTCHR enrollees who were children on 11 September 2001 for their own studies of 9/11-related physical and mental health outcomes [29,30,31,32,33,34,35,36]. They, too, were successful publishing their findings in peer-reviewed scientific journals, adding to the scientific literature on 9/11 health outcomes among children and adolescents [27].

### 4.4. Would Establishing a New Youth Cohort of Survivors Be Feasible?

The WTCHR worked with the NYC DOE to conduct a feasibility study between August 2017 and July 2020 for the creation of a new cohort of individuals exposed to the WTC attacks as children and a comparison group of unexposed youth twenty years after the event. The feasibility study sought to confirm and update address information, provided through DOE directories, and determine interest in participating in a cohort among a subset of the approximately 160,000 NYC public school students who were students in schools of lower Manhattan or selected “non-exposed” comparison areas at least 6 miles from the WTC Twin Towers in lower Manhattan on 11 September 2001 [37].

A stratified random sample of 501 youth from the exposed area (~1.5 miles from WTC site) and a comparison group of 501 youth with a similar demographic profile from the unexposed area (≥6 miles from site) were selected. Tracing involved three phases, if no response was received or mailings could not be delivered, then an individual would be moved on to the next phase of intensive tracing. From the total sample, only 3% responded to multiple mailings and expressed interest in a new child cohort. However, the demographics of respondents were skewed, with more people from the exposed zone responding than unexposed areas, along with racial/ethnic minorities exhibiting lower response rates and a higher frequency of undeliverable addresses. Scaling tracing efforts to the entire DOE population of 160,000 eligible students is estimated to cost $48 million and likely yield a sample of about 4600 former students. The overall low level of interest in participation and differential responses across several, key demographic factors indicate that constructing an epidemiologically useful cohort would require even more extensive tracing and recruitment efforts [37]. Such effort would not justify the creation of a separate children cohort given the scientific validity of the current Registry cohort of children and the uncertainty of attaining a scientifically valid new cohort.

## 5. Limitations and Strengths

Several study limitations are worth noting. First, we were unable to fully assign all the youth to census tracts that define Group 1 (Residents south of Chambers Street in Lower Manhattan) or Group 2 (between Chambers Street and Canal Street). The primary reason was that at the initial interview, resident eligibility was determined by zip code and in some cases, the address provided for residence on 11 September 2001 was not sufficient for assignment to a specific census tract that spanned several zip codes. However, there were only 12% (1795/14,656) of youth not included in groups 1 or 2 and we were still able to compare WTCHR and census using the specific census tracts for census denominator data. Second, the information on household income from the census data were not specifically for families with children and thus we could not limit representation of income to persons younger than 18 years on 11 September 2001. However, it would be assumed that the income comparison would generalize to these families with youth regardless of family composition. Third, such information as outcome rates, such as contact rate or cooperation rate, were not specifically available for residents younger than 18 years on 11 September 2001. Those rates provided in this report were for all residents, regardless of age. Fourth, because nearly 90% of children were self-identified, it may be possible that these children are different than children who were not enrolled by their parents or guardians. However, the fact that the comparison between the children in the Registry and the population indicated that they are representative suggests that self-identification was not a biasing factor.

A number of strengths of this study can be highlighted. First, with the limitations noted above we were still able to conduct detailed (at census tract level) assessments of representation by age, race/ethnic group, gender, and household income using census data for comparison. Second, we incorporated historical information on recruitment numbers and enrollment rates in addition to archival information on type of school enrollees attended. Third, we possessed basic information on youth of sufficient sample size to conduct this evaluation.

## 6. Conclusions

The WTCHR has successfully recruited and retained a sufficiently large and representative group of study participants to enable investigating health conditions among persons exposed to the 9/11 terrorist attacks as children. Although disaster studies are generally not designed to estimate population parameters, the WTCHR’s sample of 9/11 children had demographic characteristics congruent with the defined population at risk. In addition to being representative by age and sex and possessing a sufficiently large sample of sub-groups, such as Asian-Americans and low-income persons, the WTCHR children’s cohort is a unique, prospective longitudinal cohort that will be able to ascertain long-term health outcomes of a population vulnerable to disaster impact. Given that some subgroups of children may be underrepresented on the WTCHR, various analytical methods such as imputation or raking ratio estimation could be applied to offset this limitation [38]. Alternatively, forming an auxiliary WTCHR of people exposed to 9/11 as children has also been suggested to expand samples for youth research; however, a recent feasibility study concluded that the effort merited cautious deliberation given evidence of difficult recruitment and strong potential for selection bias [37]. A more practical approach would be to enumerate a new study group based on a specified research design, and only when the WTCHR sample is not sufficient to meet the aims of the research.

## Figures and Tables

**Table 1:** Source of enrollment (list or self-identified) for WTCHR residents, residents < 18 years on 11 September 2001, non-resident students, and non-resident non-students.

		Source of Enrollment			
	Total	List		Self-Identified	
	N	*n*	%	*n*	%
**Residents south of Canal St enrolled in the WTCHR**	12,861	3555	27.6	9306	72.4
**Residents < 18 years old on 11 September 2001**	1864	105	5.6	1759	94.4
Group 1: South of Chambers St ^1^	844	7	0.8	837	99.2
Group 2: North of Chambers and South of Canal Street ^2^	1020	98	9.6	922	90.4
Non-resident students < 18 years	1032	190	18.4	842	81.6
Non-resident non-students < 18 years	351	73	20.8	278	79.2
**Overall Total < 18 years in the WTCHR**	3247	368	11.3	2879	88.7

^1^ Census tracts 7, 9, 13, 15.01, 15.02, 21, and 317.01. ^2^ Census tracts 8, 16, 25, 27, 29, 31, 33, and 39.

**Table 2:** Comparison of proportions by age group of WTCHR resident children < 18 years on 11 September 2001 with 2000 census for south of Canal Street in Lower Manhattan. Denominator of proportions is Total for all residents.

Subpopulation			Age Group (Years)							
	Total (All Residents)	Total (Youth)	0–4		5–9		10–14		15–17	
	N	N	*n*	%	*n*	%	*n*	%	*n*	%
**Group 1**										
WTCHR	7460	844	321	4.3	267	3.6	204	2.7	52	0.7
Census	22,400	2390	1033	4.6	705	3.1	441	2.0	211	0.9
**Group 2**										
WTCHR	5401	1020	317	5.9	291	5.4	304	5.6	108	2.0
Census	34,779	5849	1542	4.4	1582	4.5	1736	5.0	989	2.8
Residents *										
WTCHR	12,861	1864	638	5.0	558	4.3	508	4.0	160	1.2
Census	57,179	8239	2575	4.5	2287	4.0	2177	3.8	1200	2.1

* Sum of Groups 1 and 2.

**Table 3:** Comparison of proportions for race/ethnic groups of WTCHR resident children < 18 years on 11 September 2001 with 2000 Census for south of Canal Street in Lower Manhattan. Denominator of proportions is Total for all residents.

		Race/Ethnic Group									
	Total	White		Black		Hispanic		Asian		Other	
	N	*n*	%	*n*	%	*n*	%	*n*	%	*n*	%
**Group 1**											
WTCHR	7460	547	7.3	25	0.3	88	1.2	109	1.5	75	1.0
Census	22,400	1581	7.1	82	0.4	183	0.8	377	1.7	167	0.7
**Group 2**											
WTCHR	5401	388	7.2	87	1.6	225	4.2	272	5.0	48	0.9
Census	34,779	1275	3.7	372	1.1	806	2.3	3238	9.3	158	0.5
Residents *											
WTCHR	12,861	935	7.3	112	0.9	313	2.4	381	3.0	123	1.0
Census	57,179	2856	5.0	454	0.8	989	1.7	3615	6.3	325	0.6

* Sum of Groups 1 and 2.

**Table 4:** Comparison of proportions by sex of WTCHR resident children < 18 years on 11 September 2001 with 2000 census for south of Canal Street in Lower Manhattan. Denominator of proportions is Total for all residents.

		Youth Residents			
	Total	Male		Female	
	N	*n*	%	*n*	%
**Group 1**					
WTCHR	7460	424	5.7	420	5.6
Census	44,377	1185	2.7	1205	2.7
**Group 2**					
WTCHR	5401	497	9.2	523	9.7
Census	34,779	3006	8.6	2843	8.2
Residents *					
WTCHR	12,861	921	7.2	943	7.3
Census	57,179	4191	7.3	4048	7.1

* Sum of Groups 1 and 2.

**Table 5:** Comparison of proportions by annual household income of WTCHR residents on 11 September 2001 with 2000 census for south of Canal Street in Lower Manhattan. Denominator of proportions is Total for all residents.

		Income Group (Annual Household Income)						Chi Square (*p*-Value)
	Total	<$25,000		$25,000 to $49,999		$50,000 or More		
	N	*n*	%	*n*	%	*n*	%	
WTCHR *	10,775	3106	28.8	1665	15.5	6004	55.7	171.83
**Census**	57,341	17,630	30.8	11,348	19.8	28,335	49.4	(*p* < 0.0001)

* Missing income not included in denominator.

## Data Availability

The data presented in this study are available on request from the World Trade Center Registry. The data are not publicly available due to privacy reasons.
